# Inhibitors of Phosphatidylinositol 3′-Kinases Promote Mitotic Cell Death in HeLa Cells

**DOI:** 10.1371/journal.pone.0035665

**Published:** 2012-04-24

**Authors:** Heli Hou, Yingyin Zhang, Yun Huang, Qiyi Yi, Lei Lv, Tianwei Zhang, Dawei Chen, Qiaomei Hao, Qinghua Shi

**Affiliations:** 1 School of Life Sciences, University of Science and Technology of China, Hefei, China; 2 Hefei National Laboratory for Physical Sciences at Microscale, Hefei, China; The University of Texas MD Anderson Cancer Center, United States of America

## Abstract

The phosphatidylinositol 3-kinase (PI3K) pathway plays an important role in many biological processes, including cell cycle progression, cell growth, survival, actin rearrangement and migration, and intracellular vesicular transport. However, the involvement of the PI3K pathway in the regulation of mitotic cell death remains unclear. In this study, we treated HeLa cells with the PI3K inhibitors, 3-methyladenine (3-MA, as well as a widely used autophagy inhibitor) and wortmannin to examine their effects on cell fates using live cell imaging. Treatment with 3-MA decreased cell viability in a time- and dose-dependent manner and was associated with caspase-3 activation. Interestingly, 3-MA-induced cell death was not affected by RNA interference-mediated knockdown (KD) of beclin1 (an essential protein for autophagy) in HeLa cells, or by deletion of atg5 (an essential autophagy gene) in mouse embryonic fibroblasts (MEFs). These data indicate that cell death induced by 3-MA occurs independently of its ability to inhibit autophagy. The results from live cell imaging studies showed that the inhibition of PI3Ks increased the occurrence of lagging chromosomes and cell cycle arrest and cell death in prometaphase. Furthermore, PI3K inhibitors promoted nocodazole-induced mitotic cell death and reduced mitotic slippage. Overexpression of Akt (the downstream target of PI3K) antagonized PI3K inhibitor-induced mitotic cell death and promoted nocodazole-induced mitotic slippage. These results suggest a novel role for the PI3K pathway in regulating mitotic progression and preventing mitotic cell death and provide justification for the use of PI3K inhibitors in combination with anti-mitotic drugs to combat cancer.

## Introduction

Phosphatidylinositol 3-kinases (PI3Ks) phosphorylate the 3-hydroxyl group of the inositol ring in phosphatidylinositol lipids, which in turn coordinate the localization and function of multiple effector proteins by binding to their specific lipid binding domains. At the cellular level, the PI3K pathway plays an important role in many biological processes, including cell cycle progression, cell survival, growth, migration and intracellular vesicular transport [1]. Aberrant activation of PI3Ks has been observed in a broad spectrum of human tumors [2] and is thought to confer tumors with resistance to various anti-cancer drugs and irradiation [3], [4], [5], [6].

Mitotic cell death is a mode of cell death occurring specifically during mitotic stages. Inducers of mitotic cell death include DNA damaging agents and spindle poisons/mitotic inhibitors, which activate the spindle assembly checkpoint, causing prolonged mitotic arrest and subsequent cell death during mitosis [7]. Cells that become arrested in mitosis may also slip out of mitosis due to gradual cyclinB1 degradation. This mitotic slippage may lead to the generation of tetraploid cells, which greatly restricts the use of anti-mitotic drugs in cancer treatment [8]. Thus, elucidation of the pro-death signaling pathway during prolonged mitotic arrest is important to improve the tumor-killing effects of anti-mitotic drugs. Various kinase signaling pathways have all been suggested to play a role in regulating cell death during mitotic arrest, including p38 mitogen-activated protein kinases kinase (MAPK), extracellular signal-regulated kinase (ERK), c-Jun N terminal kinase, p21-activated kinase (PAK) [9], [10], [11], and apoptosis regulators Bcl2, Bcl-xL, caspase-2/9, survivin and p73 [12], [13], [14], [15]. Inhibition of PI3Ks has been reported to sensitize tumors to the anti-mitotic drug -paclitaxel [5], [16], implying that the PI3K pathway might be involved in cell death regulation during mitotic arrest. However, additional data are needed to fully support this claim.

Autophagy is an evolutionarily conserved eukaryotic degradation pathway involved in the turnover and elimination of cellular proteins and organelles. The autophagic process is characterized by the formation of autophagosomes (double-membraned cytosolic vesicles) and subsequent lysosomal degradation of constituents contained in these vesicles [17]. Many genes involved in autophagy, including beclin1 and atg5, were initially discovered in yeast. Homologues have been identified in higher eukaryotes, and autophagy has been shown to function in various physiological and pathological processes [18], [19].

Recently reported evidence suggests the importance of autophagy in cancer development and the response to cancer treatment. 3-methyladenine (3-MA), a drug that suppresses the autophagic/lysosomal pathway by inhibiting Class III PI3Ks [20], has been widely used to study the role of autophagy in many research areas, including tumorigenesis and cancer therapy. Recently, 3-MA has been reported to cause cancer cell death under both normal and starvation conditions, which suggests that autophagy inhibitors may be useful for killing tumor cells [21], [22]. However, 3-MA could also suppress cell migration and invasion independently of its ability to inhibit autophagy, implying that 3-MA possesses functions other than autophagy suppression [22]. Thus, whether 3-MA induces cell death solely by inhibiting autophagy remains unknown.

In this study, we examined the effects of two PI3K inhibitors (3-MA and wortmannin) on mitotic cell death using live cell imaging. Our results indicate that 3-MA-induced cell death occurred independently of autophagy suppression. Live cell imaging studies demonstrated that treatment with PI3K inhibitors led to increased lagging chromosomes, prolonged arrest and significant cell death in prometaphase. Moreover, treatment with PI3K inhibitors further promoted nocodazole-induced mitotic cell death and reduced mitotic slippage. Overexpression of PI3K downstream target Akt antagonized PI3K inhibitor-induced mitotic cell death and promoted nocodazole-induced mitotic slippage. These results revealed a novel role for the PI3K pathway in preventing mitotic cell death, and provided justification for the use of PI3K inhibitors in combination with anti-mitotic drugs to improve cancer treatment outcomes.

## Results

### 3-MA induced caspase-dependent cell death that is independent of autophagy inhibition

**Figure 1:**
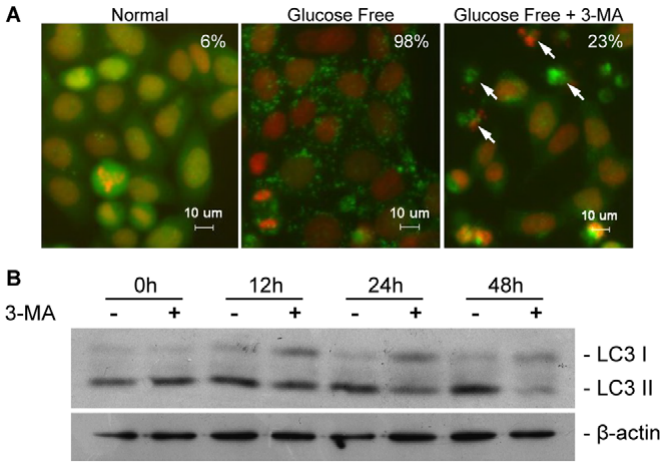
3-methyladenine (3-MA) suppressed autophagy in HeLa cells under both glucose-free conditions and normal conditions. (A) HeLa cells stably expressing GFP-LC3 were cultured in DMEM supplemented with 10% FBS (control), or in glucose-free DMEM containing 10% FBS in the absence (glucose free) or in the presence of 5 mM 3-MA (glucose free+3-MA) for 24 hours. Cells containing at least ten dots were considered to be positive for GFP-LC3 puncta. Numbers represent the percentage of GFP-LC3 puncta-positive cells and arrows indicate dead cells. At least 100 cells were counted for each treatment. (B) HeLa cells were maintained in normal cultural conditions and were treated with 5 mM 3-MA and collected at 0, 12, 24 and 48 hours post treatment initiation, and subjected to immunobloting.

First, we examined the autophagy inhibitory function of 3-MA. As shown in Fig. 1A, we examined the distribution of puncta formed by green fluorescence protein (GFP) fused with microtubule-associated light chain 3 (LC3). GFP-LC3 puncta, which are indicative of autophagy [23], were observed in 6% of HeLa cells cultured in normal culture medium and in 98% of cells cultured in glucose free medium. Treatment with 5 mM 3-MA decreased the percentage of glucose-starved HeLa cells displaying GFP-LC3 puncta to 23%. To study the role of 3-MA on autophagy under normal conditions, we treated HeLa cells with 5 mM 3-MA for 0, 12, 24 and 48 hours. As shown in Fig. 1B, the levels of LC3-I were increasing and the levels of LC3-II were decreasing between 12 and 48 hours in cells that treated with 3-MA (Fig. 1B). Thus, conversion of LC3-I to LC3-II was suppressed by 3-MA. This is consistent with the autophagy-inhibitory role of 3-MA under these conditions [23]. These results confirmed the inhibitory effects of 3-MA on autophagy under both normal and starvation conditions.

**Figure 2:**
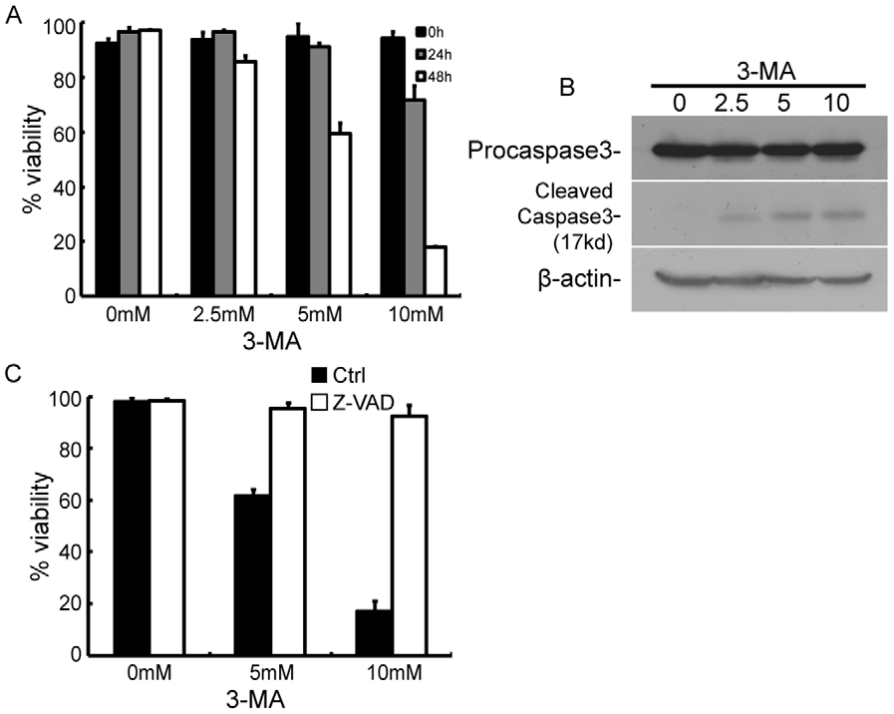
3-MA induced caspase-dependent cell death in HeLa cells. (A) HeLa cells were treated with 0, 2.5, 5 and 10 mM 3-MA. Cells were collected 0, 1 and 2 days post treatment initiation and subjected to trypan blue exclusion assay. At least 300 cells were counted for each sample. Bars represent standard deviation from three independent experiments. (B) Caspase-3 was activated upon 3-MA treatment. HeLa cells were treated with 0, 2.5, 5 or 10 mM 3-MA, and cells were collected and subjected to immunobloting 2 days post treatment initiation. Activation of caspase-3 was assessed with antibodies specific for both procaspase-3 and its active cleaved subunits. The molecular weight of cleaved subunits of caspas-3 is indicated. This blot is representative of three independent experiments. (C) z-VAD effectively antagonized 3-MA-induced cell death. HeLa cells were treated with 0, 5 or 10 mM 3-MA in the presence of 100 µM z-VAD or DMSO (used as a solvent control). Two days after treatment, the cells were collected and subjected to a trypan blue exclusion assay. At least 300 cells were counted for each sample. Bars represent the standard deviations from three independent experiments.

The effect of 3-MA on the fates of HeLa cells was then examined by trypan blue exclusion assay. As shown in Fig. 2A, treatment of HeLa cells with 2.5 mM or 5 mM 3-MA for one day did not affect cell viability, whereas treatment with 10 mM 3-MA for one day caused a 25.0% decrease in cell viability. Treatment of cells with 2.5, 5 or 10 mM 3-MA for two days caused 11.5%, 38.0% or 79.4% decrease in viability, respectively. This suggests that 3-MA decreased cell viability in a time- and dose-dependent manner (Fig. 2A).

To determine whether cell death induced by 3-MA required caspase activation, we first detected caspase-3 cleavage after 3-MA treatment. Caspase-3 is constitutively present as a 32-kD procaspase-3, which is cleaved into two active subunits of 17 kD and 12 kD upon activation [24]. As shown in Fig. 2B, treatment of cells with 2.5, 5 or 10 mM 3-MA for two days clearly promoted the cleavage of caspase-3. Addition of the pan-caspase inhibitor z-VAD at a concentration of 100 µM almost completely prevented the loss of cell viability induced 5 or 10 mM 3-MA (Fig. 2C). These results suggest that 3-MA-induced cell death is caspase dependent.

**Figure 3:**
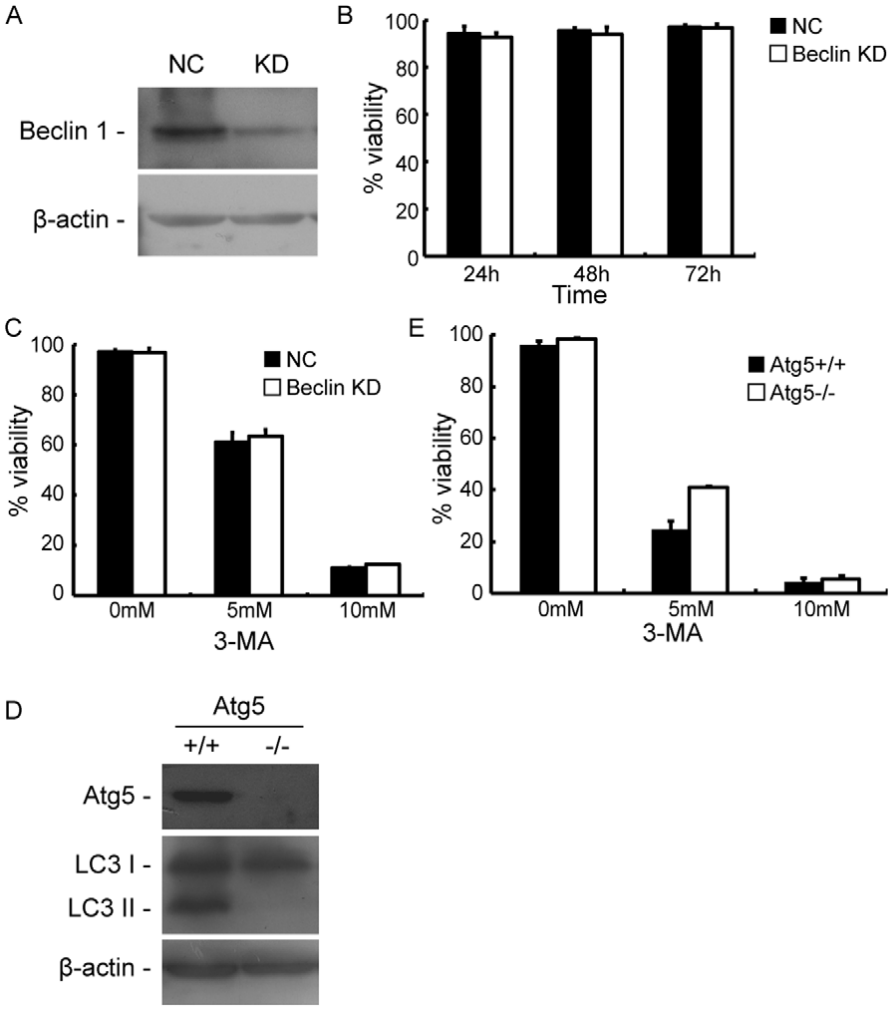
3-MA-induced cell death occurred independently of the inhibition of autophagy. (A) Immunoblot showing the efficiency of beclin1 silencing in HeLa cells. Cells were transiently transfected with negative control (NC) siRNAs or siRNAs specific for beclin1 mRNAs. The cells were collected and subjected to immunobloting 48 hours post-transfection. The blot shown is representative of three independent experiments. (B) Beclin1 KD did not cause cell death in HeLa cells. Cells were transiently transfected with negative control (NC) siRNAs or siRNAs specific for beclin1 mRNAs. Cell viability was determined using a trypan blue exclusion assay at 24, 48 and 72 hours post transfection. (C) Beclin1 KD had no effects on 3-MA-induced cell death. Twenty four hours post transfection with NC siRNAs or siRNAs against beclin1, HeLa cells were treated with 0, 5 or 10 mM 3-MA. Cell viability was determined 48 hours post treatment using trypan blue exclusion assay. (D) Immunoblot showing atg5 and LC3 levels in atg5+/+ and atg5−/− MEFs. (E) Atg5+/+ and atg5−/− MEFs were treated with 0, 5 or 10 mM 3-MA. Then cell viability were determined 48 h post treatment using trypan blue exclusion assay. At least 300 cells were counted for each treatment to determine cell viability and bars represent the standard deviations from three independent experiments.

To determine whether 3-MA-induces cell death by inhibiting autophagy, we used small interfering RNAs (siRNAs) to knock -down the expression of the autophagy protein beclin1 in HeLa cells. Silencing of beclin1 efficiently decreased the expression of its target protein (Fig. 3A). However, beclin1 KD did not affect the viability of HeLa cells. Cells transfected with siRNA specific for beclin1 did not show a significant decrease in viability when compared to cells transfected with non-specific control siRNA at 24, 48 or 72 hours post transfection (Fig. 3B). Furthermore, beclin1 KD did not enhance the lethal effect of 3-MA. As shown in Fig. 3C, siRNA-transfected HeLa cells treated with 5 mM and 10 mM 3-MA for two days displayed 33.3% and 84.4% decreases in viability, respectively. These results were similar to the effects observed in cells transfected with control siRNA (35.8% and 86.0% decreases in viability, respectively).

To further determine whether 3-MA could induce cell death in autophagy-deficient cells, we examined atg5−/− MEFs, which do not express LC3-II, and thus completely lack the capacity for autophagy (Fig. 3D). As shown in Fig. 3E, treatment with 5 mM and 10 mM 3-MA for two days decreased the viability of atg5−/− MEFs by 57.2% and 92.8%, respectively. This decrease in cell viability was significantly different from that observed in atg5+/+ MEFs (71.5% and 91.6% decreases in viability, respectively). Collectively, these results indicate that 3-MA induces cell death independently of its ability to inhibit autophagy.

### Inhibitors of PI3Ks induced both interphase and mitotic cell death

**Figure 4:**
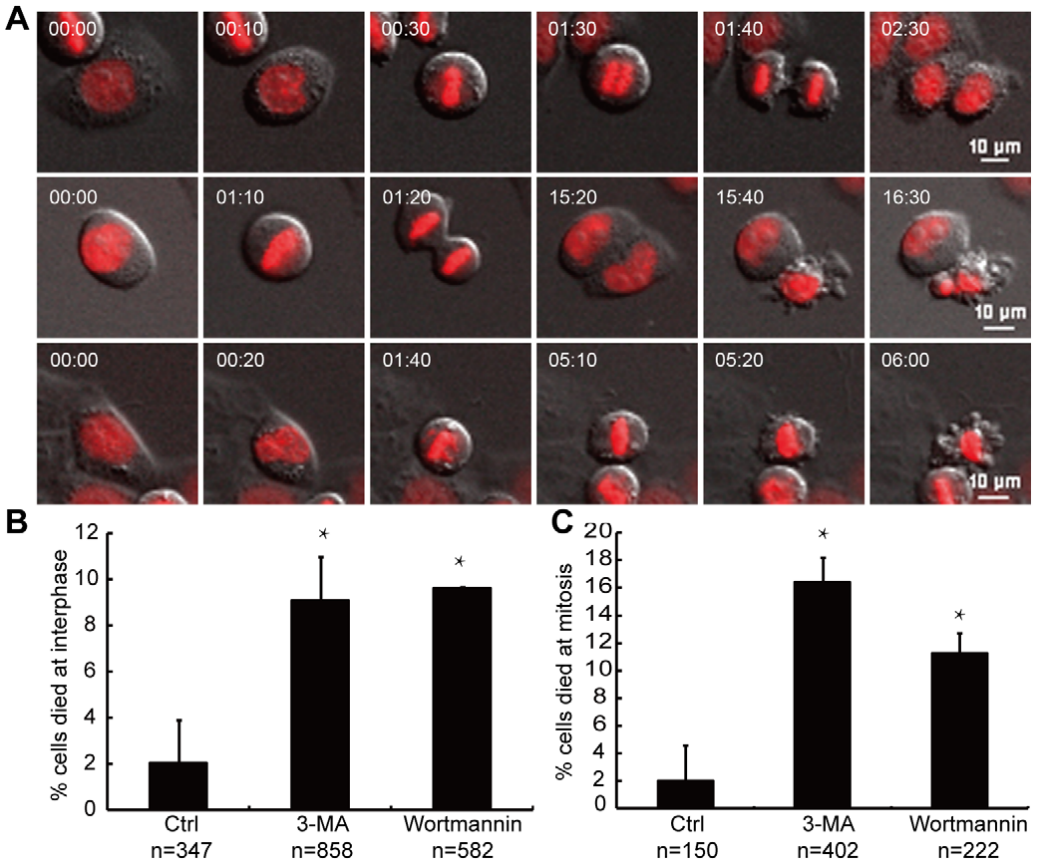
PI3K inhibitors induced cell death in both interphase and mitosis. HeLa cells were stably transfected with mCherry tagged histone-2B to visualize the nuclei. The cells were treated with 5 mM 3-MA or 50 µM wortmannin, and live cell imaging was performed for 48 hours. (A) Representative live cell imaging records of normal mitotic process (top panel), interphase cell death (middle panel) and mitotic cell death (bottom panel). Time is shown as hours: minutes. (B) Quantification of 3-MA or wortmannin induced interphase cell death. (C) Quantification of 3-MA- or wortmannin-induced mitotic cell death. n: the number of cells analyzed. *: p<0.001, 2×2 χ^2^ test, compared to the control. Error bars represent the standard deviations from two independent experiments.

PI3Ks are the only reported targets for 3-MA [22]. To determine whether 3-MA-induced cell death was dependent on PI3K inhibition and to examine the modes of cell death induced by 3-MA, we treated HeLa cells with another PI3K inhibitor, wortmannin, and subsequently performed long-term live cell imaging to examine their behaviors. During normal mitosis (Fig. 4A, top panel; Movie S1), chromatin became condensed and congressed onto the metaphase plate during prometaphase. This was followed by chromosomal segregation and decondensation to form two daughter nuclei during anaphase and telophase. The entire mitotic process, from prophase to telophase, lasted approximately 2.5 hours in HeLa cells. Treatment of cells with PI3K inhibitors induced cell death during both interphase and mitosis. For cells that died in interphase (Fig. 4A, middle panel; Movie S2), their mother cells usually underwent mitosis and produced two daughter cells with one daughter died before entering the next round of mitosis. For cells that died in mitosis (Fig. 4A, bottom panel; Movie S3), the mother cell rounded up with the chromatin beginning to condense and congress to form a metaphase plate, indicating that the cell was in prometaphase. It stayed in prometaphase for about six hours before the signs of apoptotic cell death appeared, including wrinkling of the plasma membrane, collapse of the cytoplasm and the condensation or fragmentation of the nuclei. As shown in Fig. 4B and C, 9.1% and 16.4% of cells died in interphase and mitosis, respectively, following 5 mM 3-MA treatment, and 9.6% and 11.3% of cells died in interphase and mitosis, respectively, after 50 µM wortmannin treatment. The frequency of cell death during mitosis or interphase was significantly higher than that observed in the control cells (interphase: 2.0%, p<0.001; mitosis: 2.0%, p<0.001; 2×2 χ^2^ test). These results indicate that inhibitors of PI3K induced cell death in both interphase and mitosis.

### Inhibitors of PI3K promoted prometaphase chromosome lagging and prolonged the duration of prometaphase

**Figure 5:**
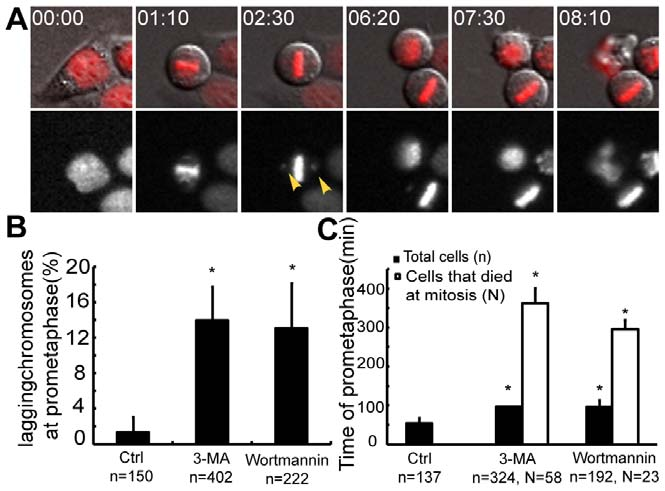
Inhibitors of PI3K increased chromosome lagging and prolonged the duration of prometaphase. HeLa cells were treated with 5 mM 3-MA or 50 µM wortmannin, and live cell imaging was performed for 48 hours. (A) Representative live cell imaging records showing a cell that stayed at prometaphase for more than eight hours (top panel), with the presence of lagging chromosomes outside the metaphase plate (bottom panel). (B) Quantification of 3-MA- or wortmannin-induced lagging chromosomes at prometaphase. n: the number of total cells. *: p<0.001, 2×2 χ^2^ test, compared to the control. (C) Quantification of the duration of prometaphase under 3-MA or wortmannin treatment. n: the number of total cells. N: the number of cells that died in mitosis. *: p<0.001, student's t-test, compared to the control. Error bars represent standard deviations from the results of two independent experiments. Time is shown as hours: minutes.

Mitotic cell death has been reported to occur after prolonged mitotic arrest [7]. Using live cell imaging to record the mitotic behaviors of single cells, we assessed the ability of PI3K inhibitors to cause mitotic arrest. We noticed that cells often stayed in prometaphase for several hours without entering anaphase before dying in mitosis (Fig. 5A, top panel; Movie S4). The average duration of prometaphse was significantly prolonged in cells treated with 5 mM 3-MA or 50 µM wortmannin (96.6 min for 3-MA; 96.2 min for wortmannin; Fig. 5C), when compared to control cells (53.8 min, p<0.001, student's-t test; Fig. 5C). The duration of prometaphase was even longer for cells that died in mitosis (361.8 min for 3-MA; 295.7 min for wortmannin; Fig. 5C). Thus, PI3K inhibitor-treated cells showed a prolonged prometaphase before undergoing cell death.

Lagging chromosomes that do not align onto the metaphase plate could activate the spindle assembly checkpoint and cause prolonged prometaphase [25]. We thus closely examined the behaviors of chromosomes during mitosis, and found that chromosomal laggards often lingered outside the metaphase plate, even several hours after mitotic entry (Fig. 5A, bottom panel; Movie S5). 13.9% of 3-MA treated cells and 13.1% of wortmannin-treated cells displayed lagging chromosomes at prometaphase, as compared to 1.3% of control cells (Fig. 5B).

### PI3K inhibitors promoted nocodazole induced mitotic cell death and reduced mitotic slippage

**Figure 6:**
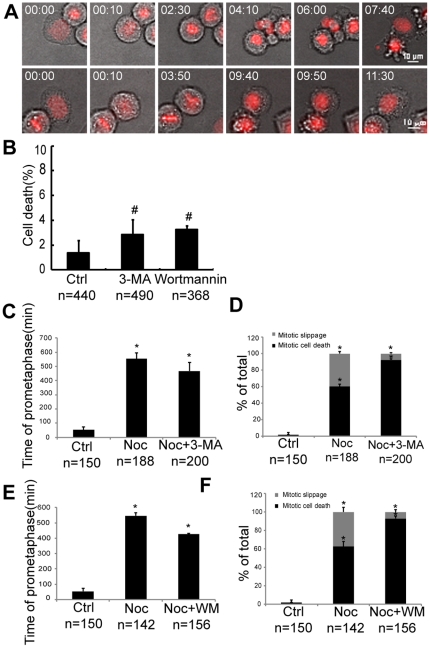
PI3K inhibitors promote nocodazole-induced mitotic cell death and reduce mitotic slippage. HeLa cells were treated with 100 nM nocodazole alone or in combination with 1 mM 3-MA or 10 µM wortmannin, and live cell imaging was performed for 48 hours. (A) Representative live cell imaging records showing nocodazole-induced mitotic cell death (top panel) and mitotic slippage (bottom panel). (B) Quantification of 1 mM 3-MA or 10 µM wortmannin induced cell death. #: p>0.05, 2×2 χ^2^ test, compared with the control. (C&E) The duration of prometaphase for cells treated with 100 nM nocodazole alone or in combination with 1 m M 3-MA or 10 µM wortmannin. *: p<0.001, student's t-test, compared with the control. (D&F) The frequencies of mitotic cell death for cells treated with 100 nM nocodazole alone or in combination with 1 mM 3-MA or 10 µM wortmannin. *: p<0.001, 2×2 χ^2^ test, compared to the control. n: The number of cells analyzed. Error bars represent the standard deviations from two independent experiments. Time is shown as hours: minutes.

The duration of prometaphase before Hela cells died in mitosis was approximately five to six hours after treatment with PI3K inhibitors (Fig. 5C). This timeframe was much shorter than that of cells treated with classic anti-mitotic drugs such as nocodazole [26], [27], [28]. This implies that PI3K inhibition may potentially accelerate the process of mitotic cell death. To confirm this finding, we treated HeLa cells with nocodazole, a classic anti-mitotic drug, in combination with 3-MA or wortmannin and examined cell death using live cell imaging. After treatment with 100 nM nocodazole, approximately 40% of cells exhibited mitotic slippage, while the remainder exhibited mitotic cell death (Fig. 6D and F). For those exhibited mitotic cell death, the cell entered mitosis and stayed in mitosis for approximately eight hours without forming a metaphase plate and then committing to death (Fig. 6A, top panel; Movie S6). For those cells that exhibited mitotic slippage, the cell entered mitosis and stayed in mitosis for greater than ten hours, then decondensed its chromosomes without undergoing anaphase, finally forming one daughter cell in interphase (Fig. 6B, bottom panel; Movie S7).

We next treated cells with 1 mM 3-MA or 10 µM wortmannin alone, or in combination with 100 nM nocodazole and examined cell death using live cell imaging. Treatment of HeLa cells with 1 mM 3-MA or 10 µM wortmannin alone did not cause significant cell death (Fig. 2A and 6B). However, 3-MA significantly shortened the duration of nocodazole-induced-prometaphase arrest (from 546.2 min to 426.4 min, p<0.001; student's t test, Fig. 6C) and reduced the occurrence of nocodazole-induced mitotic slippage (from 41.2% to 8.8%, p<0.001; 2×2 χ^2^ test, Fig. 6D). Similar results were obtained with wortmannin treatment (Fig. 6E and F). These results indicate that PI3K inhibition promoted nocodazole-induced mitotic cell death and reduced mitotic slippage.

### Akt overexpression antagonized PI3K inhibitor-induced mitotic cell death and promoted nocodazole-induced mitotic slippage

**Figure 7:**
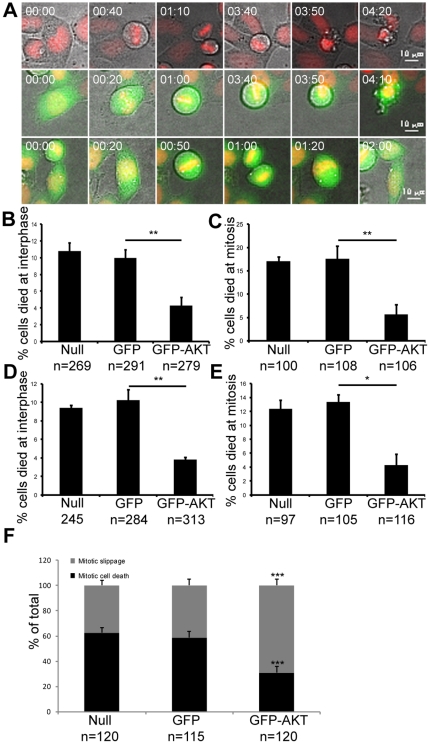
Akt overexpression antagonized PI3K inhibitor-induced mitotic cell death and promoted nocodazole induced mitotic slippage. HeLa cells were transfected with plasmids expressing GFP or GFP-Akt and were treated with 5 mM 3-MA, 50 µM wortmannin or 100 nM nocodazole and subjected to live cell imaging 24 hours post transfection. (A) Representative live cell imaging records showing the daughter cells of a cell that was not transfected with GFP that underwent interphase cell death (top panel), a GFP expressing cell that underwent mitotic cell death (middle panel) and a GFP-AKT expressing cell that underwent normal mitosis (bottom panel) in the presence of PI3K inhibitors. (B&D) Frequencies of interphase cell death in cells treated with 5 mM 3-MA or 50 µM wortmannin. (C&E) Frequencies of mitotic cell death in cells treated with 5 mM 3-MA or 50 µM wortmannin. (F) Frequencies of mitotic slippage in cells treated with 100 nM nocodazole. Null: cells that were not transfected with any vectors; GFP: cells transfected with GFP expressing vectors; GFP-AKT: cells transfected with GFP-AKT expressing vectors. *: p<0.05, **: p<0.01, ***: p<0.001, 2×2 χ^2^ test, compared to the control. Error bars represent the standard deviations from two independent experiments. Time is shown as hours: minutes.

3-MA or wortmannin may possess off-target effects other than inhibition of PI3Ks; thus, we transiently expressed a constitutively active form of Akt in HeLa cells to test whether Akt overexpression could reverse the effects of PI3K inhibitors on cell death. Transfection of HeLa cells with a control vector harboring GFP had little effect on 3-MA- or wortmannin-induced cell death (Fig. 7B, C, D and E). However, expression of GFP-Akt significantly reduced both interphase cell death and mitotic cell death induced by treatment with 5 mM 3-MA (interphase: from 10.0% to 4.3%, p<0.01; mitosis: from 17.6% to 5.7%, p<0.01; 2×2 χ^2^ test; Fig. 7B and C). Similar results were obtained with 50 µM wortmannin (interphase: from 10.2% to 3.8%, p<0.01; mitosis: from 13.3% to 4.3%, p<0.05, 2×2 χ^2^ test, Fig. 7D and E).

The effects of Akt overexpression on cell death in nocodazole treated cells were also investigated. As expected, expression of GFP itself had little effect on nocodazole-induced mitotic slippage (Fig. 7F). However, expression of GFP-Akt construct significantly increased the occurrence of nocodaozle-induced mitotic slippage (from 41.4% to 69.1%, p<0.001, 2×2 χ^2^ test; Fig. 7F). Taken together, these results indicate that Akt overexpression antagonized PI3K inhibitor-induced mitotic cell death and promote nocodazole-induced mitotic slippage.

## Discussion

3-MA is a widely used inhibitor of autophagy, and it has been reported to cause HeLa cell death under both normal and starvation conditions, leading to the hypothesis that autophagy inhibitors may be useful for killing tumor cells [21], [22]. In this study, we consistently found that 3-MA increased HeLa cell death in a time- and dose-dependent manner (Fig. 2A). However, beclin1 down-regulation did not induce HeLa cell death, nor did it affect 3-MA-induced cell death (Fig. 3B and C). Moreover, 3-MA treatment induced significant cell death in autophagy-deficient atg5−/− MEFs (Fig. 3E). These results indicated an autophagy-independent inhibitory function of 3-MA in inducing cell death. Thus, special cautions should be taken when interpreting the results obtained with similar types of autophagy inhibitors. Notably, a significant difference in cell viability was observed between atg5+/+ and atg5−/−MEFs when treated with 5 mM 3-MA (Fig. 3E). This might be due to the apoptosis-promoting function of atg5 [29].

Because PI3Ks are the only reported targets for 3-MA [22], we used another PI3K inhibitor (wortmannin) to treat HeLa cells and tracked cell death using live cell imaging. Consistent with previous reports [4], [30], inhibition of PI3Ks was observed to cause cell death in interphase. We found that inhibition of PI3Ks induced cell death during mitosis and that overexpression of the PI3K downstream target Akt antagonized PI3K inhibitor-induced mitotic cell death. Live cell imaging studies further showed that PI3K inhibitors induced prometaphase chromosome lagging and prolonged the duration of prometaphase. These results revealed a novel role for the PI3K pathway in regulating cell cycle progression during mitosis and preventing mitotic arrest.

Mitotic cell death is defined as a mode of cell death that occurs during mitosis. Various anti-mitotic drugs have been shown to induce cell death during mitosis. These drugs include taxanes, Vinca alkaloids and kinesin inhibitors, which interfere with the functions of mitotic spindle apparatus, DNA damaging agents, which activate the spindle assembly checkpoint, or other treatments that prevent mitotic exit through mechanisms such as CDC20 down-regulation [26], [31]. In this study, we found that PI3K inhibitor-treated cells frequently displayed lagging chromosomes at prometaphase (Fig. 5A and B). This implies that the microtubule-kinetochore attachment may be impaired in cells treated with PI3K inhibitors, thus activating the spindle assembly checkpoint and causing mitotic arrest and cell death during mitosis. Disruption of microtubule-kinetochore attachments has been shown to cause mitotic cell death. Depletion of hNuf2, a kinetochore protein essential for microtubule attachment, induced mitotic arrest and subsequently mitotic cell death [32]. Furthermore, expression of a dominant negative Plk1, which are involved in microtubule-kinetochore attachment, caused mitotic cell death in HeLa cells [33], [34]. Whether PI3K inhibition-induced mitotic cell death involves one of these proteins or other unknown factors remains to be determined.

Mitotic cell death may occur in a caspase-dependent or -independent manner. Inhibition of Chk2 in syncytia generated by fusion of asynchronous HeLa cells caused mitotic cell death accompanied by sequential caspase-2 activation, cytochrome C release from mitochondira, caspase-3 activation and DNA fragmentation [35]. Anti-mitotic drugs, including nocodazole, taxol or kinesin-5 inhibitor, have also been shown to cause mitotic cell death mediated by caspase activation [27], [36]. However, in bub1 deficient cells, conditions that activate the spindle checkpoints (i.e., cold shock or treatment with nocodazole, paclitaxel, or 17-AAG) induced caspase-independent mitotic death and required apoptosis-inducing factor and endonuclease G [37]. In this study, treatment with PI3K inhibitors activated caspase-3, and the pan-caspase inhibitor z-VAD almost completely antagonized PI3K inhibitor-induced cell death (Fig. 2B and C). The results of live cell imaging studies showed that PI3K inhibitor-treated cells displayed signs of apoptosis, including wrinkled plasma membrane, collapsed cytoplasm and condensed or fragmented nuclei. These results indicate that 3-MA-induced mitotic cell death occurred through caspase-dependent apoptosis.

The underlying trigger for mitotic cell death during prolonged mitotic arrest is currently unclear. Spindle assembly checkpoint (SAC) has long been thought to play critical roles during this process. A recent study showed that silencing of SAC proteins did not affect the mitotic arrest or mitotic cell death induced by down-regulation of CDC20 or expression of degradation-resistant cyclin B1 [26]. This leads to the suggestion that some general features of mitotic arrest, rather than SAC itself, are the proximal trigger for death during mitosis. Nevertheless, the molecular nature of the signal that triggers cell death during prolonged mitotic arrest remains poorly defined. PI3K inhibitors have also been reported to sensitize tumor cells to antimitotic drugs including paclitaxel [5], [16], indicating that the PI3K pathway may be involved in cell death regulation during mitotic arrest. However, concrete evidence supporting this conclusion is lacking. In this study we demonstrated by live cell imaging that inhibitors of PI3K prolonged the duration of prometaphase which was followed by death during mitosis. Notably, PI3K inhibitor-treated HeLa cells stayed in mitosis for only five to six hours on average before they committed to cell death (Fig. 5A and C), and this cell death occurred much sooner than the mitotic cell death induced by conventional anti-mitotic drugs. It has been reported that most HeLa cells stay in mitosis for more than ten hours before death induced by treatment with nocodazole or kinesin5 inhibitors [26], [27], [28]. This suggests that inhibition of PI3K may promote cell death during mitotic arrest. Treatment of HeLa cells with PI3K inhibitors in combination with nocodazole promoted mitotic cell death and reduced mitotic slippage, and Akt overexpression increased the occurrence of nocodazole-induced mitotic slippage (Fig. 7). These results directly demonstrated that the PI3K-Akt pathway plays an important role in preventing mitotic cell death.

It is interesting to note that we found PI3K inhibitors increased the duration of prometaphase when used alone (Fig. 4C), whereas these inhibitors decreased the time of prometaphase required to initiate nocodazole-induced cell death (Fig. 6C and E). These results suggest that the PI3K pathway plays multiple roles in regulating mitotic cell death. When used alone, PI3K inhibitors induced lagging chromosomes and caused cell cycle arrest at prometaphase (Fig. 5). Certain pro-death signals may accumulate during this arrest, thus leading to mitotic cell death. When used in combination with nocodazole, PI3K inhibitors shortened the time required to initiate nocodazole-induced cell death and reduced the occurrence of mitotic slippage (Fig. 6). This implies that PI3Ks act as a pro-survival pathway during mitotic arrest, which may confer tumor cells with resistance to anti-mitotic drugs.

Classic anti-mitotic drugs induce cancer cell death mainly through the activation of SAC and by increasing mitotic arrest and mitotic cell death. However, cancer cells often slip out of mitotic arrest before cell death due to defective SAC or gradual proteolysis of cyclinB1, which reduces the efficacy of conventional anti-mitotic drugs [8]. Elucidation of the pro-death signaling pathway during prolonged mitotic arrest is important to improve the tumor killing effects of anti-mitotic drugs. In this study, we demonstrated that inhibition of PI3Ks promoted nocodazole-induced mitotic cell death and reduced mitotic slippage. This finding suggests that using PI3k inhibitors in combination with anti-mitotic drugs may improve cancer treatment outcomes.

In summary, the current study demonstrated that the inhibition of PI3K pathway induced mitotic arrest and mitotic cell death and promoted nocodazole-induced mitotic cell death while reducing the occurrence of mitotic slippage. These results suggest a novel role for the PI3K pathway in regulating cell cycle progression during mitosis and preventing mitotic cell death, and provide justification for the use of PI3K inhibitors in combination with anti-mitotic drugs to combat cancer.

## Materials and Methods

### Cell lines and treatment

HeLa cells and MEF atg5−/−, atg5+/+ cells (a generous gift from Dr. Noboru Mizushima, Tokyo Metropolitan Institute of Medical Science) were cultured in DMEM supplemented with 10% fetal bovine serum and 1% non-essential amino acids (Invitrogen). H2B-mCherry-positive or GFP-LC3-positive HeLa cells were obtained as follows. Cells were grown in a 24-well plate (3×10^4^ cells per well) for 24 hours and transfected with pBOS-H2BmCherry (mCherry expressing vector was a generous gift from Dr. Chenbei Chang, University of Alabama at Birmingham) or pEGFP-LC3 (kindly provided by Dr. Longping Wen, University of Science a nd Technology of China) using Lipofectamine 2000 transfection reagent (Invitrogen 11668-027). Thirty hours after transfection, the cells were inoculated into 60-mm tissue culture dishes and selected with 2 µg/ml blasticidin S (MP Biomedicals, 150477) or 500 µg/ml G418 (GIBCO, 11811-031) for 2 or 3 weeks. All incubations were performed at 37°C in a humidified atmosphere of 5% CO_2_ and 95% air.

3-MA, which was found to inhibit autophagy at concentrations ranging from 1 to 10 mM [20], was purchased from Sigma Aldrich (Cat. No. 08592), and was directly dissolved into the culture medium at the indicated concentrations. Wortmannin (Beyotime, S1952), nocodazole (Calbiochem, 487928) and z-VAD (Calbiochem, 627610) were dissolved in DMSO and diluted in culture medium.

### Cell viability assay

Cell viability was determined by a trypan blue exclusion assay. Briefly, both adherent and floating cells were collected and suspended in phosphate buffered saline (PBS, pH 7.4) at a final density of 1–2×10^6^/ml. An equal volume of 0.4% trypan blue solution (w/v, in PBS) was added to the cell suspension and mixed thoroughly. After incubation at room temperature for 3 min, cell counting was performed using a hemacytometer.

### Live cell imaging

Cells were seeded in an 8-well coverglass-bottomed chamber (Lab-Tek II, Cat No. 155409) for 24 hours (6×10^3^ cells per well). Images were acquired automatically at multiple locations on the coverglass using a Nikon TE2000E inverted microscope fitted with a 20× Nikon Plan Apo objective, a linearly-encoded stage (Proscan, Prior) and a Hamamatsu Orca-ER CCD camera. A mercury-arc lamp with two neutral density filters (for a total 128-fold reduction in intensity) was used for fluorescence illumination. The microscope was controlled using NIS-Elements Advanced Research software (Nikon) and housed in a custom-designed 37°C chamber with a secondary internal chamber that delivered humidified 5% CO_2_. Fluorescence and differential interference contrast images were obtained every 10 min for a period of 48 hours.

To analyze live cell imaging movies, the time-lapse records of live cell imaging experiments were exported as an image series, and analyzed manually using NIS-Elements Advanced Research software (Nikon). The criteria for analyses were described previously [38], and lagging chromosomes in prometaphase were defined as the red fluorescence-positive materials that lingered outside the roughly formed metaphase plate for more than 3 frames (30 min).

### RNA interference

siRNAs targeting beclin1 mRNA (5′- CAGUUUGGCACAAUCAAUAUU -3′) and nonspecific control siRNAs (GenePharma, Shanghai) were used in the following experiments. Transfection was performed using Lipofectamine 2000 (Invitrogen 11668-027). One day prior to transfection, the cells were plated at an appropriate density to grow to 70%–80% confluency overnight. siRNA-Lipofectamine 2000 complexes were then prepared, and transfection was performed according to the manufacturer's instructions. The cells were incubated with siRNA-liposome complexes for 6 hours and then provided with fresh DMEM containing 10% FBS.

### Transient transfection of HeLa cells with GFP and GFP-Akt expression vectors

HeLa cells that had reached 70%–80% confluency were transfected with pLEGFP-N1 and pLEGFP-AKT (a generous gift from Dr. Yizheng Wang, Chinese Academy of Science) using Lipofectamine 2000 (Invitrogen 11668-027) according to the manufacturer's instructions. Twenty-four hours post-transfection, the cells were seeded into an 8-well coverglass-bottomed chamber (Lab-Tek II, Cat No. 155409) for additional treatment.

### Western blotting

Antibodies specific for Beclin1 (Santa Cruz, SC11427), Atg5 (a generous gift from Dr. Hans-Uwe Simon, University of Bern), LC3 (Novus Biologicals, NB100-2220), Caspase3 (Beyotime, AC031) and β-actin (Abcam, ab8226) were used. Western blot analysis was performed as described previously (25). Proteins were extracted from HeLa cells with lysis buffer (50 mM Tris (pH 7.4), 150 mM NaCl, 1% NP-40, 0.5% sodium deoxycholate, 0.1% SDS and 1 mM PMSF). Equal amounts of protein (20 µg) were separated by sodium dodecyl sulfate-polyacrylamide gel electrophoresis (SDS-PAGE) (12% separating gel). After electrophoresis, the proteins were transferred to nitrocellulose membranes (200 mA for 3 hours). The blots were then blocked in 5% nonfat dry milk solution for 1 hour at room temperature.The membrane were incubated with the respective primary antibodies at room temperature and then with anti-rabbit or anti-mouse alkaline phosphatase-conjugated secondary IgG antibodies. Immune complexes were detected with an enhanced chemiluminescence detection method by immersing the blots in chemiluminescence reagents (Pierce 34150) for 5–10 min and then exposing the blotsto Kodak X-OMAT film for a few seconds.

### Statistical analysis

The Student's-t test was used to compare continuous variables and the chi-squared (2×2χ2) test was used to compare categorical variables. The p-values were as shown and less than 0.05 was considered statistically significant.

## Supporting Information

Movie S1 **Normal cell division.** The cell rounded up and the chromatins became condensed and congressed onto the metaphase plate during prometaphase. The chromosomes were then segregated and decondensed to form two daughter nuclei during anaphase and telophase, generating two daughter cells.
(AVI)

Movie S2 **Interphase cell death.** The cell underwent mitosis and produced two daughter cells. One daughter cell died before entering the next round of mitosis.
(AVI)

Movie S3 **Mitotic cell death.** The cell rounded up as the chromatin began to condense and congress to form a metaphase plate, then the cell died without entering anaphase.
(AVI)

Movie S4 **Prolonged duration in prometaphase.** This cell entered mitosis and stayed in mitosis for many frames (10 min/frame).
(AVI)

Movie S5 **Lagging chromosomes.** This cell entered mitosis but stayed in prometaphase, with the red fluorescent-positive materials observed outside the roughly formed metaphase plate.
(AVI)

Movie S6 **Nocodazole induced mitotic cell death.** The cell entered mitosis and stayed in mitosis for a prolonged period without forming a metaphase plate before committing to death.
(AVI)

Movie S7 **Nocodazole-induced mitotic slippage.** The cell entered mitosis and stayed in mitosis for a prolonged period, and then decondensed its chromosomes without undergoing anaphase. One daughter cell was finally formed in interphase.
(AVI)
